# Selection of Endogenous Control Reference Genes for Studies on Type 1 or Type 2 Endometrial Cancer

**DOI:** 10.1038/s41598-020-64663-4

**Published:** 2020-05-21

**Authors:** Thangesweran Ayakannu, Anthony H. Taylor, Justin C. Konje

**Affiliations:** 1 Faculty of Health and Life Sciences, University of Liverpool, Thompson Yates Brownlow Hill, Liverpool, L69 3GB UK; 2 Reproductive Sciences Section, Department of Cancer Studies and Molecular Medicine, University of Leicester, Leicester, UK; 3 Gynaecology Oncology Cancer Centre, Liverpool Women’s NHS Foundation Trust, Liverpool Women’s Hospital, Liverpool, UK; 4 Department of Molecular and Cell Biology, University of Leicester, Leicester, Leicestershire UK; 5 Department of Obstetrics and Gynaecology, Sidra Medicine, Women’s Wellness and Research Center, HMC, Doha, Qatar

**Keywords:** Biophysical methods, Endometrial cancer

## Abstract

A panel of 32 candidate reference genes was used to identify the most stable genes for gene normalisation in quantitative RT-PCR studies using endometrial biopsies obtained from women with endometrial cancer (type 1 or type 2) and without cancer (controls). RNA from the biopsies was isolated, examined for purity and quality, and then reverse transcribed into cDNA before being subjected to real-time qRT-PCR analysis in triplicate within the TaqMan gene Expression Assay kit. The most ‘stable’ endogenous control genes were then identified using the geNorm qbase + 2 and NormFinder software packages. PSMC4, PUM1 and IPO8 were identified as the best reference genes combination for type 1 endometrial cancer (grades 1, 2 and 3), whereas for type 2 endometrial cancer (serous and carcinosarcoma), UBC, MRPL19, PGK1 and PPIA were the best reference genes combination. We conclude that the use of these normaliser combinations should provide accurate interpretation of gene expression at the transcript level in endometrial cancer studies especially for types 1 and 2 cancers.

## Introduction

The use of biomarkers is the cornerstone of effective precision medicine^1,2^. RNA forms an excellent source of biomarkers to enable early disease detection, assessment of prognosis, monitoring patient response to therapy or selecting the right treatment for the right patient (personalised medicine). The gold standard method for studying biomarkers at the RNA level in the past decade has been measurement of microRNA in plasma^3^ and in the past two decades measurement of messenger RNA (mRNA) in tissue biopsies^4,5^. The measurement of these moieties has occurred primarily through quantitative real time PCR (qRT-PCR) with microRNA- and gene-specific primers, respectively. Application of qRT-PCR to the study of transcript levels in many disease processes has increasingly replaced northern blotting because it is easy to use, fast, reproducible, highly sensitive, specific, and provides high sample throughput^6–9^. In particular, it has been used to identify and assess several molecular markers associated with the staging^10^, initiation^11^, progression^12^, and metastatic potential^13^ of endometrial cancer and recently in patient prognosis^14,15^.

Elimination of sources of error in the qRT-PCR technique, such as differences in the quantity and quality of extracted mRNA, the presence of contaminating genomic or operator DNA, divergences in reverse transcription and PCR efficiencies must occur for the qRT-PCR to be valid^16–18^. To ensure uniformity and reproducibility of published data, the “Minimum Information for publication of Quantitative real time PCR Experiments” (MIQE) guidelines suggest that the choice of and number of reference genes should be an essential part of all qRT-PCR studies. Validation of this important step guarantees normalisation of resulting data^19^. This normalisation step, where an endogenous reference (housekeeping) gene compensates for any variations in sample or experimental conditions, is essential because all genes under test (both the genes of interest and the reference gene) are assessed under the same experimental conditions. A unified, single “best” reference gene is therefore unlikely to be found routinely, because almost all genes are modified under some conditions^20^.

In earlier qRT-PCR studies of endometrial cancer, normalisation was done using reference genes, such as β-actin (ACTB)^21,22^, glyceraldehyde-3-phosphate dehydrogenase (GAPDH)^23,24^, or 18S RNA ribosomal unit 1 (18S rRNA)^25^, chosen without rigorous testing. Previously, we demonstrated that all of the aforementioned genes are poor reference genes for the study of gene expression changes that occur in the two main types of endometrial cancer^5^. In our previous studies, we showed that a combination of 3 genes (IPO8, MRPL19 and PPIA) provided the best combination of normalisation factors in qRT-PCR studies of endometrial cancer and that geNorm qbase+2^26^ is more robust than the other software packages in its statistical corrections for all the possible sources of experimental error listed above. Although this conclusion remains applicable and supportable when comparing mRNA levels in type 1 endometrial cancer with type 2 endometrial cancer in the same gene expression study, recent studies have identified additional targeted biomarkers for individualised endometrial cancer. In those studies, the authors advocate the linking of transcript studies directly to personalised treatments, without indicating what the correcting reference genes for such studies should be^13^. This is especially important because endometrial cancer is becoming more prevalent in the reproductive age woman^10^, a point that has been missed in those recommendations^1,12^.

The aim of this study was therefore to identify the best reference gene combinations for the normalisation of studies, when using qRT-PCR, to quantify the number of gene transcripts in endometrial tissues from normal women with either endometrial cancer type 1 or type 2. By doing so, we hope to provide investigators with the tools to effectively investigate their chosen RNA biomarkers in future studies of women with type 1 or type 2 endometrial cancer so as to provide meaningful clinical data, especially when only one of these patient groups is under consideration. If both groups are under consideration, then our previously published data remains the correct choice^4^.

## Results

### GeNorm analyses for type 1 EC and for type 2 EC

**Figure 1:**
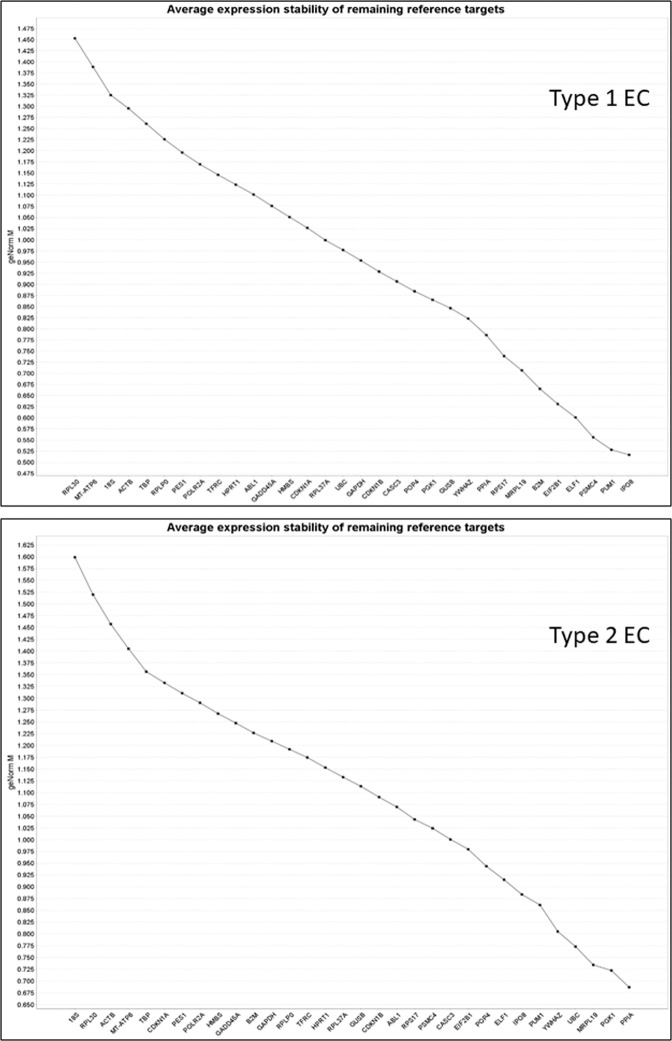
GeNorm analyses of the reference genes. Average expression stability values (geNorm M) of the 32 reference genes are ranked from least to most stable expression (left to right) for type 1 EC data (upper panel) and type 2 EC data (lower panel). Data are presented according to the output file obtained from qbase + 2 software. An M value of ≤ 1.0 indicates more stable gene expression.

Using geNorm, the least stable to most stable reference genes evaluated for type 1 EC was found to be: RPL30 > MT-ATP6 > 18S > ACTB > TBP > RPLP0 > PES1 > POLR2A > TFRC > HPRT1 > ABL1 > GADD45A > HMBS > CDKN1A > RPL37A > UBC > GAPDH > CDKN1B > CASC3 > POP4 > PGK1 > GUSB > YWHAZ > PPIA > RPS17 > MRPL19 > B2M > EIF2B1 > ELF1 > PSMC4 > PUM1 > IPO8 (Fig. 1); (gene names, identities, accession numbers and amplicon sizes of the PCR products can be found in Supplemental Table 1). Analysis of this order indicated that PUM1 and IPO8 were the two most stable genes (defined as M ≤ 1.0) in type 1 EC samples, with the two least stable genes being RPL30 and MT-ATP6. The commonly used reference genes β-actin (ACTB), GAPDH and 18S were outside the least stable (M ≥ 1.0) category with M-values of 1.355, 0.950 and 1.300, respectively.

**Figure 2:**
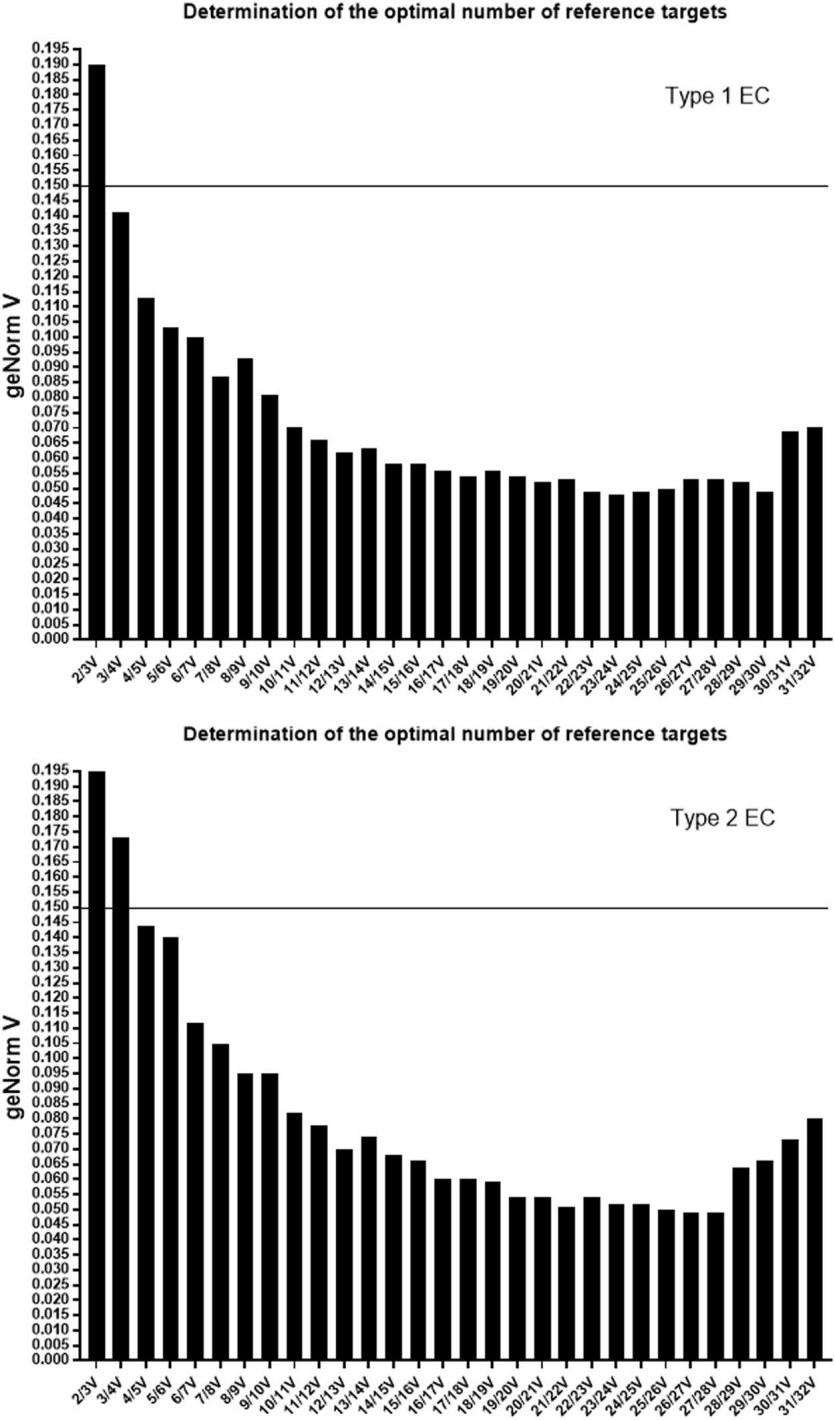
Determination of the optimal number of reference genes for normalisation. geNorm V scores, using the pair-wise variation (*V*) analyses within the geNorm^PLUS^ analysis software for the 32 reference genes for the incremental combination of V2/3 to V31/32 genes, are shown. The upper panel shows the data output file obtained for control and type 1 EC samples and the lower panel equivalent output data for control and type 2 EC samples. The horizontal line indicates the position of the geNorm threshold for stability (V = 0.15).

Gene stability analysis using geNorm (Fig. 2) indicated that the optimal number of reference gene targets was 5 in the EC1 analyses (geNorm V < 0.15 when comparing a normalisation factor based on the 5 or 6 most stable targets). Thus, geNorm qbase + 2 predicts that the optimal normalisation factor would be the geometric mean of the reference targets PSMC4, PUM1 and IPO8 (based on 3 most stable genes), ELF1, PSMC4, PUM1 AND IPO8 (based on 4 most stable genes), or EIF2B1, ELF1, PSMC4, PUM1 AND IPO8 (based on 5 most stable genes).

Similar analyses of samples from patients with type 2 EC demonstrated the least stable to most stable genes to be: 18S > RPL30 > ACTB > MT-ATP6 > TBP > CDKN1A > PES1 > POLR2A > HMBS > GADD45A > B2M > GAPDH > RPLP0 > TFRC > HPRT1 > RPL37A > GUSB > CDKN1B > ABL1 > RPS17 > PSMC4 > CASC3 > EIF2B1 > POP4 > ELF1 > IPO8 > PUM1 > YWHAZ > UBC > MRPL19 > PGK1 > PPIA (Fig. 1). In studies using normal and type 2 cancer endometria, PGK1 and PPIA would be the two most stable genes with 18S and RPL30 being the two least stable. The commonly used reference genes (β-actin (ACTB), GAPDH and 18S) were also outside the least stable category with M-values of 1.450, 1.205 and 1.600, respectively.

The optimal number of reference targets for type 2 EC, was also 5 genes (Fig. 2). Thus, geNorm^PLUS^ with qbase+2 predicts that optimal normalisation factor would be achieved by using the geometric mean of the reference targets MRPL19, PGK1 and PPIA (based on 3 most stable genes), UBC, MRPL19, PGK1 and PPIA (based on 4 most stable genes), or YWAZ, UBC, MRPL19, PGK1 and PPIA (based on 5 most stable genes).

### NormFinder analyses for type 1EC and for type 2 EC

**Figure 3:**
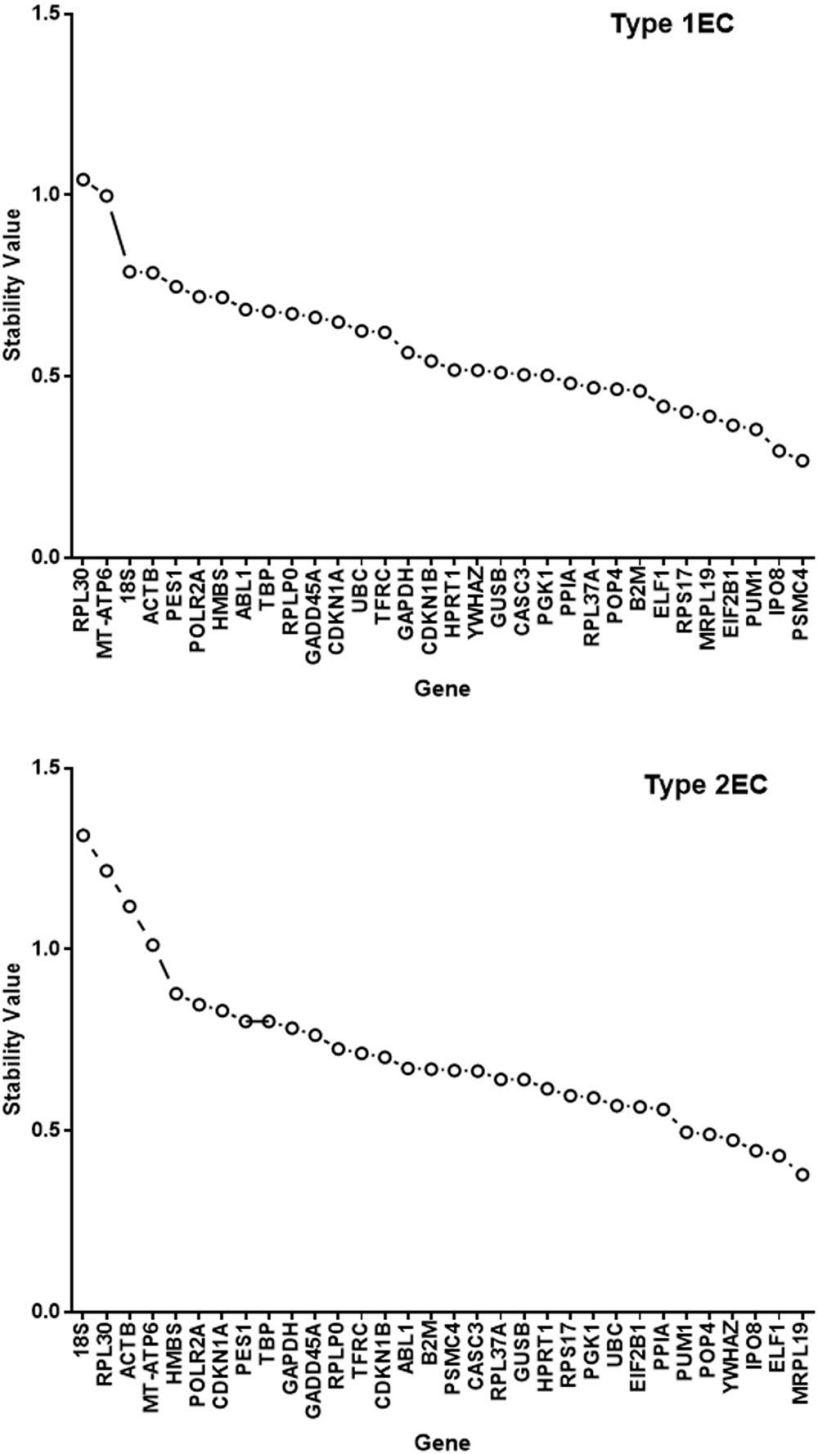
Normfinder analyses of the reference genes. The stability values for each of the 32 ‘housekeeping’ genes are shown, with the least stable on the left and the most stable on the right. A stability value < 0.5 is sufficient to predict either a stable gene in the type 1 EC cohort (upper panel) or the type 2 EC cohort (lower panel).

NormFinder software, which uses a mathematical model that considers both intergroup and intragroup expression variations (stability), and ranks them in order from the lowest to highest stability value^27^, identified PSMC4 (proteasome (prosome, Macropain) 26S subunit, ATPase, 4) as the single most stable gene (stability value = 0.268). The software specified that the best two gene combination was IPO8 (importin 8) and MRPL19 (mitochondrial ribosomal protein L19*)* with a stability value of 0.224 for patients with type 1 EC (Fig. 3). By contrast, the single most stable gene for type 2 EC patients was MRPL19 (mitochondrial ribosomal protein L19; stability value of 0.379) and the best combination of two genes was ELF1 (E74-like factor 1) and PUM1 (Pumilio homolog 1) with a stability value of 0.259 (Fig. 3).

## Discussion

In this first systematic study, we show which combination of reference genes from a panel of 32 endogenous control reference genes should be used for normalisation in quantitative real-time PCR (qRT-PCR) studies of types 1 and 2 EC. We have identified 2 sets (one for type 1 and another set for type 2) of five ‘housekeeping’ genes that provide the best combination. When the TaqMan gene Expression Assay kit was used, none of the genes in the two identified combinations was common to both types of EC. When the stability list was increased to six normalising genes, one gene (PUM1) was found to be common to both sets of samples and interestingly one gene (IPO8) in the type 1 EC group and two genes (PPIA and MRPL19) in the type 2 EC group were identical to those we identified in our previous report^5^ where only 3 normalising genes were used and in other cancers where 4 normalising genes were used^28^. Several genes (B2M, EIF2B1, ELF1 and PSMC4; type 1 EC, and YWAZ, UBC, and PGK1; type 2 EC) were not identified in the combinations of five best genes for the two cancer sub-types (this study) suggesting that the transcriptome of these two EC sub-types may in fact be very different. Indeed, type 1 EC is phenotypically distinct from type 2 EC both in its biology and response to treatment, further supporting our hypothesis that the normalising genes for these two subtypes of EC would also be different; qRT-PCR studies must therefore take this into consideration in order to generate meaningful and clinically relevant data. The data in this new analysis suggest that the best normalisers for qRT-PCR studies that are confined to either type 1 EC or type 2 EC alone are different hence combining these together when studying either in isolation is likely to generate erroneous data.

These findings have important implications for future study design, because qRT-PCR studies that only focus on type 1 EC (with a control group) require a different combination of normalising genes to a study that focusses on only type 2 EC (also with a control group). If the study design utilises type 1 and type 2 EC samples (along with a control of normal tissues), then an additional and different set of normalising genes will be required to provide valid and important data, as previously described^5^. For example, in a study of only type 1 EC samples, the list of normalising genes required for qRT-PCR studies are those listed in the results section of this paper, whilst a study of all types of EC requires the list of genes provided previously^5^, i.e. PPIA, MRPL19 and IPO8. The 3 normalising genes we reported previously was not significantly improved upon by increasing the list to 4, 5 or 6 normalising genes^5^ and so this extra expense is not required, nor warranted.

Using NormFinder to analyse the best combination of normalising genes and the best gene for each patient group revealed that two genes previously reported to be the best normalisers for studies that included all types of EC (i.e. IPO8 and MRPL19)^5^ were also identified in the present study. Furthermore, MRPL19 appears to be the only common gene in the combination of six that is most stable in studies separately investigating type 1 EC and type 2 EC (as in this study), and indeed is one of the genes we have previously recommended when both types of EC were combined^5^.

Previous studies focussing on the best normalising genes for studies of patients with endometrial cancer have a limitation in that they evaluated only ten possible normalising genes^29,30^. In those studies, the best normalising gene lists identified genes that were identified here and also previously (PPIA) in type 2 EC, but not those identified in type 1 EC. An additional issue in the stated studies was the large number of samples being examined that was not balanced by an equivalent number of control samples^30^. Increasing the number of biological sample replicates minimises, any internal variations. This could result in erroneous conclusions based on those internal limitations. By limiting our studies to the minimum number of biological replicates, we have maximally increased the biological and experimental variations. This means that the software needs to perform in a robust manner to generate the best normalisers for the study populations. Additionally, by increasing the number of ‘housekeeping genes’ from ten to 32 genes, we have increased the probability of identifying extra most stable normalisers for qRT-PCR studies, not identified previously. Consequently, we also reported the minimum number of genes (n = 3) for each type of endometrial cancer and the maximum number of genes (n = 6) for absolute robustness. The decision on whether to choose 3, 4, 5 or 6 genes in combination as normalisers for qRT-PCR studies, is dependent on two factors. These factors are: (1) the magnitude of change in transcript levels (more subtle changes need a larger number of genes to robustly identify them) and (2) cost (the cost of six primer pairs is obviously more than that of 3, 4 or 5). These considerations are important because an unwise choice may invalidate the study undertaken.

The use of the correct endogenous control reference gene(s) for normalisation in qRT-PCR experiments has been the subject of strong debate^31^; with advantages and disadvantages^17^, even with studies that follow MIQE guidelines^19^ being highlighted. Nevertheless, studies have shown disparities in the stability of reference genes in many different tissues^32^ and in the same tissues under different conditions^33^. It is thus imperative that the appropriate selection of stable reference genes relative to the experiment undertaken is made, as has been discussed previously^5,30^. In this regard, improved normalisation is possible when changing from one to multiple endogenous control reference genes, a single reference is unlikely to provide an ideal endogenous control, as is stated in the MIQE guidelines. It is for this reason that we chose to undertake this study, especially as advocated personalised medicine studies do not define the subject samples or the experimental conditions when suggesting new biomarkers for EC. We also deliberately chose samples from a cross section of the different groups that truly represent the patient population. By using both pre- and post-menopausal samples and also limiting our study to 3 patient samples from each group to provide nine control samples, nine type 1 EC samples and six type 2 EC samples that were clearly defined by an experienced gynaecology histopathologist, we maximised the variation within and between the sample groups. This manipulation results in the most robust study where variation is being minimised, whereas a much larger number of samples (e.g. in the tens or hundreds) would have hidden the variation that is needed by the software being used.

It is for this reason that we recommended geNorm qbase + 2 software for the analysis of normalisation studies where a given combination of reference genes are used to generate a normalisation factor and that the data are further complemented with analysis of the same data using NormFinder^34,35^. By providing the best possible combination of normaliser genes, a platform whereby the biology of type 1 EC or type 2 EC can effectively be studied at the transcript level is provided. Additionally, in patient-specific prognosis studies where treatment outcomes are assessed, the observations reported herein are instantly applicable, since it provides a good starting point for normalising gene identification when treatments are applied. Thus, in gene expression studies using normal and type 1 malignant endometria, and where limited amounts of material or resources are available, more reliable normalisation is achieved when using the geometric mean of the Ct values obtained from the combination of three genes of PSMC4, PUM1 and IPO8 and is thus recommended. Similarly, for type 2 EC studies the geometric mean of the Ct values derived from the combination of MRPL19, PGK1 and PPIA provides a reliable normalisation factor. For absolute robustness, we recommend the use of geometric means from the 5 genes EIF2B1, ELF1, PSMC4, PUM1 AND IPO8 for type 1 EC studies and the use of geometric means from the 5 genes YWAZ, UBC, MRPL19, PGK1 and PPIA for type 2 EC studies, especially if the changes in gene expression when compared to controls are relatively subtle in contrast to other highly expressed gene targets. To compare gene expression patterns between type 1 EC and type 2 EC samples (without any control tissues as a reference), researchers are advised to follow the protocols described in this and our previous publication^5^, to generate the best normalising genes for their own patient cohorts.

In summary, by using a panel of 32 optimised and validated endogenous control reference genes in a Taqman gene expression assay format, we identified the most robust endogenous control reference genes for the study of either type 1 EC or type 2 EC. In doing so, we can categorically remove traditionally used normalising genes for the study of either type 1 EC or type 2 EC by qRT–PCR from the database, since the array used included representative genes from different gene families and functional classes. By reporting these data, we hoped to have provided a valuable tool for use in future studies of RNA biomarkers in the biology of type 1 and type 2 EC.

## Materials and Methods

All volunteers provided signed, written informed consent to partake in the study (see Ethics Statement section). The Leicestershire, Northamptonshire and Rutland Research Ethics Committee (ref 06/Q2501/49) approved the study. Women undergoing hysterectomy for endometrial carcinoma or a benign gynaecological condition at the University Hospitals of Leicester National Health Service Trust were recruited (Table 1).

**Table 1:** Patient characteristics.

Control Patients	Age (yrs)	Mean ± SD	p-value^†^	Mean ± SD for Control Patients	p-value^††^	BMI (kg/m^2^)	Mean ± SD	p-value^†^	Mean ± SD for Control Patients	p-value^††^
Atrophic (1)	65					34				
Atrophic (2)	65	62.33 ± 4.62	n.a.			22	26.67 ± 6.43	n.a.		
Atrophic (3)	57					24				
Secretory Phase (1)	44					25				
Secretory Phase (2)	51	46.00 ± 4.36	**0.025**	51.89 ± 5.477	n.a.	27	26.00 ± 1.00	n.s.	26.22 ± 3.38	n.a.
Secretory Phase (3)	43					26				
Proliferative Phase (1)	47					27				
Proliferative Phase (2)	48	47.33 ± 0.58	**0.037**			27	26.00 ± 1.73	n.s.		
Proliferative Phase (3)	47					24				
**Cancer Patients (all Stage 1)**	Age (yrs)	Mean ± SD	p-value†	Mean ± SD for Cancer Patients	p-value^††^	BMI (kg/m^2^)	Mean ± SD	p-value^†^	Mean ± SD for Cancer Patients	p-value^††^
EC1 Grade 1 (1)	63					33				
EC1 Grade 1 (2)	88	78.00 ± 13.23	**0.030**			24	31.33 ± 6.66	n.s.		
EC1 Grade 1 (3)	83					37				
EC1 Grade 2 (1)	78					29				
EC1 Grade 2 (2)	69	67.67 ± 11.06	0.936	72.78 ± 11.43	**0.0002**	32	30.67 ± 1.53	n.s.	32.44 ± 5.08	**0.012**
EC1 Grade 2 (3)	56					31				
EC1 Grade 3 (1)	74					42				
EC1 Grade 3 (2)	60	72.67 ± 12.06	0.486			30	35.33 ± 6.11	**0.038**		
EC1 Grade 3 (3)	84					34				
EC2 Serous (1)	61					40				
EC2 Serous (2)	55	59.00 ± 3.46	0.994			35	37.67 ± 2.52	**0.011**		
EC2 Serous (3)	61					38				
EC2 Carcinosarcoma (1)	50			54.50 ± 6.25	0.817	34			37.17 ± 4.40	**0.0002**
EC2 Carcinosarcoma (2)	45	50.00 ± 5.00	0.317			44	36.67 ± 6.43	**0.019**		
EC2 Carcinosarcoma (3)	55					32				

^†^P-value compared to atrophic patient group; ^††^P-value compared to control group; One-way ANOVA with Dunnett’s multiple comparisons t-test. Significant differences are in bold font. EC1 = endometrial carcinoma type 1; EC2 = endometrial carcinoma type 2; n.a. = not applicable; n.s. = not significantly different; S.D. = standard deviation.

Histological diagnosis of the cancer was based on the FIGO system^36^ and 15 endometrial carcinoma samples were studied: type 1 grade 1 endometrioid adenocarcinoma (n = 3), type 1 grade 2 endometrioid adenocarcinoma (n = 3) and type 1 grade 3 endometrioid adenocarcinoma (n = 3), type 2 serous (n = 3) and carcinosarcoma (n = 3). All cancer tissues was classified as being FIGO Stage 1. Normal endometrial tissue samples were obtained from volunteers who were undergoing a hysterectomy for benign indications (prolapse, dysfunctional uterine bleeding, fibroids) and were classified into pre-menopausal (secretory (n = 3) or proliferative (n = 3) phase) and postmenopausal demonstrating histological atrophic endometria (n = 3) by the criteria of Noyes *et al*.^37^. Patient characteristics are shown in Table 1.

The type 1 EC patients (72.8 ± 11.4 years; Mean ± SD) were significantly older (p < 0.001; one way ANOVA with Dunnett’s multiple comparison test) than either the controls (51.9 ± 8.5 years) or those with type 2 EC (54.5 ± 6.2 years). Both type 1 (32.4 ± 5.1 kg/m^2^) and type 2 EC (37.2 ± 4.4 kg/m^2^) patients had significantly higher BMIs (p < 0.01; one way ANOVA with Dunnett’s multiple comparison test) when compared to the controls (26.2 ± 3.4 kg/m^2^), as has been reported previously^38^ (Table 1).

### Preparation of total cellular RNA and cDNA synthesis

From this point forward, the methodologies are essentially the same as described^4,5^. Fresh uteri were transported on ice to the histopathology department and two adjacent tissue biopsy samples dissected out by an experienced consultant gynaecology histopathologist using a dissecting microscope; one sample was fixed in 10% formal saline, stained with haematoxylin and eosin and used for histological confirmation of the diagnosis. All cancer samples were stage 1 and all control tissues were devoid of myometrial contamination. The second sample used for this normalising gene study was washed with phosphate buffered saline (PBS) to remove excess blood and stored in RNAlater (Life Technologies, Paisley, UK) for 24 hours at room temperature before transfer to −80 °C for storage and further processing.

Frozen, endometrial tissues (~100 mg) in lysis/binding buffer (1 ml lysis/binding buffer solution per 100 mg of tissues (miRNA Isolation Kit) were homogenised using a TissueRuptor (Qiagen Crawley, UK) homogeniser at medium speed for 60 seconds on ice until all visible ‘clumps’ were dispersed. Total RNA was extracted using the mirVana™ miRNA Isolation Kit (Life Technologies, Paisley, UK) according to the manufacturer’s protocol. Total RNA was then quantified and its purity determined using a NanoDrop 2000c spectrophotometer (Thermo Scientific, Detroit USA). At this point, the RNA concentration was standardised to 10 μg/100 μl, and contaminating genomic DNA digested by treating with a TURBO-DNAse free kit (Life Technologies, Paisley, UK) at 37 °C for 30 minutes. The reaction was inactivated with 10 μl of inactivation buffer and the solution centrifuged for 90 seconds at 10 000 x g. The purity of the extracted total RNA (supernatant), as measured with the Nanodrop spectrophotometer, indicated good quality RNA with a A_260_/A_280_ ratio of 2.10 ± 0.31 (OD ratio ± SD) and a A_260_/A_230_ ratio of 2.19 ± 0.43. The average nucleic acid yield of RNA after extraction was 1.17 ± 0.61 (μg/μl ± SD). The treated supernatants were subjected to first strand synthesis using the high capacity cDNA MultiScribe™ Reverse Transcriptase Kit ((Life Technologies, Paisley, UK) according to the manufacturer’s protocol with 1 μg of RNA, oligodT16 primers and recombinant MMLV-RT enzyme; incubation at 25 °C for 10 minutes, 37 °C for 120 minutes, 85 °C for 5 minutes and then cooled to 4 °C. The resultant cDNA was stored at −20 °C.

### qRT-PCR studies

Quantitative Real-Time PCR experiments were performed in triplicate using optimised, validated human endogenous control assay TaqMan Array 96-Well Plates (Applied Biosystems by Life Technologies, Paisley, UK) that contain 32 reference genes (see Supplemental Table 1). TaqMan Gene Expression Assays were used (Applied Biosystems) and each consisted of a fluorogenic FAM dye–labelled MGB probe (final concentration 250 nM) and two amplification primers (forward and reverse; final concentration 900 nM) provided in a pre-formulated 20X mix. Each assay had an amplification efficiency of 100 ± 10%, calculated by the system software. RT-minus and no template controls (NTC) containing DNAse-free water instead of template mRNA were included in each run and no product was synthesised in the NTC and RT-minus reactions confirming the absence of contamination with exogenous DNA. The final reaction volume was 20 μl and consisted of 2 μl of cDNA, 8 μl of DNAse-free water and 10 μl of TaqMan universal PCR Master Mix. A StepOne Plus instrument (Applied Biosystems by Life Technologies, Paisley, UK) was used for the PCR with their proprietary software (StepOnePlus software version 2.3) automatically determining the Ct as being 0.5 standard deviations above baseline fluorescence. The cycle profile was 2 minutes at 50 °C, 10 minutes at 95 °C, 40 cycles of 15 seconds at 95 °C and 1 minute at 60 °C.

### Determination of the most stable reference genes

Gene stability was evaluated using geNorm^PLUS^ software v2.2 (Biogazelle, Zwijnaarde, Belgium), which incorporates the updated version of qbase + 2^26^ (available from https://www.biogazelle.com/qbaseplus) and NormFinder version 0.953^27^ (available from Aarhus University, Denmark; http://moma.dk/normfinder-software). Accumulated standard deviation data obtained from the NormFinder algorithm were analysed using GenEx software version 5.3.6.170 (MultiD Analyses AB, Goteborg, Sweden) (http://www.multid.se/contact.php).

Briefly, mRNA gene expression stability analysis for the 32 endogenous reference control genes was obtained using the mean qRT-PCR threshold cycle (Ct) value, defined as the number of cycles required for the fluorescent signal to cross the threshold (i.e. 0.5 standard deviations above background levels). The geNorm qbase+2 algorithm determined the medium reference target stability measure (M), as the average pair-wise variation of each reference gene in relation to all the other reference genes thus enabling the elimination of the least stable gene. This was followed by recalculation of the M values resulting in a ranking of the most stable genes, i.e. the lower the M value, the higher the gene stability. The software indicates that a stable reference gene should have an average geNorm M value ≤ 1.0 in a heterogeneous set of samples^26^.

The in-built geNorm qbase + 2 ranking algorithm was used to calculate the minimum number of genes required for a reliable normalisation factor, using the pair-wise variation Vn/Vn + 1 between two sequential normalisation factors (NF_n_ and NF_n+1_). In addition, using the in-built optimal cut off value (V = 0.15) for pairwise variation, below which the inclusion of an additional reference genes is unnecessary, the minimum number of reference gene for type 1 EC studies or type 2 EC studies was calculated^26^.

Details of the statistical methods used by both qbase + 2 and NormFinder are described elsewhere^39–41^.

### Ethics statement

All subjects gave written informed consent in accordance with the Declaration of Helsinki. The study and the protocol adopted was approved by the Leicestershire, Northamptonshire and Rutland Research Ethics Committee (LREC Reference Number: 06/Q2501/49) and performed using guidelines provided within the National Health Service Research Governance Framework.

## Supplementary information

Supplemental Table.
