# Supplementary material for: Selection of Endogenous Control Reference Genes for Studies on Type 1 or Type 2 Endometrial Cancer
Source: Sci Rep. 2020 May 21;10:8468. doi: 10.1038/s41598-020-64663-4 (PMC7242460; doi:10.1038/s41598-020-64663-4)
Supplement: Supplementary file 1 — Supplemental Table. [file 41598_2020_64663_MOESM1_ESM.docx]

**Selection of Endogenous Control Reference Genes for Studies on**

**Type 1 or Type 2 Endometrial Cancer**

**by**

Thangesweran Ayakannu*, Anthony H. Taylor and Justin C. Konje

Supplemental Table 1. TaqMan Endogenous Control Genes

| **Gene ID** | **Gene name** | **Accession number** | **Life Technologies assay ID** | **Amplicon size (bp)** |
| --- | --- | --- | --- | --- |
| 18S | Eukaryotic 18S ribosomal RNA | X03205 | Hs99999901_s1 | 187 |
| ACTB | Actin, beta | NM_001101 | Hs99999903_m1 | 171 |
| ABL1 | V-abl Abelson murine leukemia viral oncogene homolog 1 | NM_005157 | Hs00245445_m1 | 91 |
| B2M | Beta-2-microglobulin | NM_004048 | Hs99999907_m1 | 75 |
| CASC3 | Cancer susceptibility Candidate 3 | NM_007359 | Hs00201226_m1 | 67 |
| CDKN1A | Cyclin-dependent kinase Inhibitor 1A (p21, Cip1) | NM_000389 | Hs00355782_m1 | 66 |
| CDKN1B | Cyclin-dependent kinase Inhibitor 1B (p27, Kip1) | NM_004064 | Hs00153277_m1 | 71 |
| EIF2B1 | Eukaryotic translation initiation factor 2B, subunit 1 alpha,26kDa | NM_001414 | Hs00426752_m1 | 75 |
| ELF1 | E74-like factor 1 (ets domain transcription factor) | NM_001145353 | Hs00152844_m1 | 76 |
| GADD45A | Growth arrest and DNA- damage-include, alpha | NM_001199741 | Hs00169255_m1 | 123 |
| GAPDH | Glyceraldehyde-3-phosphate dehydrogenase | NM_002046 | Hs99999905_m1 | 122 |
| GUSB | Glucuronidase, beta | NM_000181 | Hs99999908_m1 | 81 |
| HMBS | Hydroxymethylbilane synthase | NM_000190 | Hs00609297_m1 | 64 |
| HPRT1 | Hypoxanthine phosphoribosyl transferase | NM_000194 | Hs99999909_m1 | 100 |
| IPO8 | Importin 8 | NM_006390 | Hs00183533_m1 | 71 |
| MRPL19 | Mitochondrial ribosomal protein L19 | NM_014763 | Hs00608519_m1 | 72 |
| MT-ATP6 | Mitchondrially encoded ATP synthase 6 | NC_012920 | Hs02596862_g1 | 150 |
| PES1 | Pescadillo homolog 1, containing BRCT domain (zebra fish) | NM_001243225 | Hs00362795_g1 | 56 |
| PGK1 | Phosphoglycerate kinase | NM_000291 | Hs99999906_m1 | 75 |
| POLR2A | Polymerase (RNA) II (DNA-directed) polypeptide A | NM_000937 | Hs00172187_m1 | 61 |
| POP4 | Processing of precursor 4, ribonuclease P/MRP subunit (S. cerevisiae) | NM_006627 | Hs00198357_m1 | 64 |
| PPIA | Peptidylpropyl isomerase A (cyclophilin A) | NM_021330 | Hs99999904_m1 | 98 |
| PSMC4 | Proteasome (prosome, Macropain) 26S subunit, ATPase, 4 | NM_006503 | Hs00197826_m1 | 83 |
| PUM1 | Pumilio homolog 1(Drosophila) | NM_ 001020658 | Hs00206469_m1 | 89 |
| RPL37A | Ribosomal protein L37a | NM_000998 | Hs01102345_m1 | 125 |
| RPL30 | Ribosomal protein L30 | NM_000989 | Hs00265497_m1 | 149 |
| RPLPO | Ribosomal protein, large, PO | NM_001002 | Hs99999902_m1 | 105 |
| RPS17 | Ribosomal protein S17 | NM_001021 | Hs00734303_g1 | 93 |
| TBP | TATA box–binding protein | M34960 | Hs99999910_m1 | 127 |
| TFRC | Transferrin receptor (P90, CD71) | NM_003234 | Hs99999911_m1 | 105 |
| UBC | Ubiquitin C | NM_021009 | Hs00824723_m1 | 71 |
| YWHAZ | Tyrosine 3-monooxygenase/tryptophan 5-monooxygenase activation protein, zeta polypeptide | NM_003406 | Hs00237047_m1 | 70 |

**Details obtained from http://thermofisher.com; bp = basepair**
